# Supplementary material for: A linear tessellation model for the identification of “food desert”: A case study of Shanghai, China
Source: PLoS One. 2025 Jan 22;20(1):e0317003. doi: 10.1371/journal.pone.0317003 (PMC11753656; doi:10.1371/journal.pone.0317003)
Supplement: Attachment — Submitted filename: Response to Reviewers.docx [file pone.0317003.s001.docx]

Responses

We thank the editors and reviewers for their valuable requirements and comments, which have enabled us to significantly improve this paper. We carefully revised the manuscripts and corresponding figures. We chose the track changes mode in Word to highlight our changes in the manuscripts, where the texts in blue and strikethrough refers to the deleted contents, and the texts in red and underline refers to the added contents. The original Figures 1 to 12 have been modified and renumbered as Figures 1 to 15.

Please find our responses to each individual requirement and comment below.

Comments from the Editors and Reviewers:

Editors

Requirements to the Author

Please use the space provided to explain your answers to the questions above. You may also include additional comments for the author, including concerns about dual publication, research ethics, or publication ethics. (Please upload your review as an attachment if it exceeds 20,000 characters)

The responses to all comments are as follows:

Journal requirements:

**1.** **Please ensure that your manuscript meets PLOS ONE's style requirements, including those for file naming.**

Thank you for this requirement.

We have revised the title page, manuscripts, figures and other submitted files according to PLOS ONE's style requirements. And we uploaded a separate document in “.doc” format containing all the modified figures, and uploaded each revised figure in “.tif” format in the document named “Figures.rar” as Supporting Information.

We hope you are satisfied with all the modifications.

**2. We note that you have stated that you will provide** **repository information for your data at acceptance. Should your manuscript be accepted for publication, we will hold it until you provide the relevant accession numbers or DOIs necessary to access your data. If you wish to make changes to your Data Availability statement, please describe these changes in your cover letter and we will update your Data Availability statement to reflect the information you provide.**

Thank you for this reminder.

We have provided repository information for our data in the form of private link. When our manuscript is accepted, we will change the private link to a public link for readers to browse and download our experimental data.

We hope you are satisfied with this operation.

**3. Please include a separate caption for each figure in your manuscript.**

Thank you for your requirement.

We have added the separate caption for subgraphs in each figure, as seen in the document named “Figure.doc” and the submitted figures in “.tif” format in the document named “Figures.rar”.

We hope you are satisfied with the modifications about all the figures.

**4. We note that Figures 1, 2, 6, 9, 10, 11 and 12 in your submission contain [map/satellite] images which may be copyrighted. All PLOS content is published under the Creative Commons Attribution License (CC BY 4.0), which means that the manuscript, images, and Supporting Information files will be freely available online, and any third party is permitted to access, download, copy, distribute, and use these materials in any way, even commercially, with proper attribution. For these reasons, we cannot publish previously copyrighted maps or satellite images created using proprietary data, such as Google software (Google Maps, Street View, and Earth). For more information, see our copyright guidelines: http://journals.plos.org/plosone/s/licenses-and-copyright.We require you to either (1) present written permission from the copyright holder to publish these figures specifically under the CC BY 4.0 license, or (2) remove the figures from your submission.**

Thank you for this reminder.

In our manuscripts, all the figures are created by Lu Wang who is the first author of this paper. Therefore, she is the copyright holder of Figures 1, 2, 6, 9, 10, 11 and 12. We have sought permission to use the figures 1, 2, 6, 7, 8, 9, 12, 13, 14 and 15 from her. And we uploaded the document named “Permission form.pdf” as a submission file labeled with the file type “Other” in new submission.

**5. We note that your author list was updated during the revision process. Please complete our Authorship Change Form by following this link: http://journals.plos.org/plosone/s/file?id=13d0/plos-one-change-to-authorship-form.docx If you are adding or removing more than 2 authors, you can complete multiple forms and submit as many forms as needed to reflect the updates. Please return the form(s) as an attachment by emailing plosone@plos.org or by uploading it as a submission file labeled with the file type ‘Other’. Please note that if your manuscript is accepted, we will not be able to complete the publication process without the completed form.**

Thank you for your reminder.

We have uploaded the document named “Request for change to authorship.pdf” as a submission file labeled with the file type “Other” in new submission.

**6. Please note that PLOS ONE is unable to publish previously copyrighted maps or satellite images, or images created using proprietary data. For these reasons, we cannot publish images generated by software which copyrights their output (such as Google Maps, Street View, and Earth). In order to use these images in your submission, we require explicit permission from the copyright owner to publish the figures under the CC BY 4.0 license.**

**At this time, please kindly clarify the following regarding Figures 1, 2, 6, 7, 8, 9, 12, 13, 14 and 15:**

**a) Where did the authors obtain the [maps, satellite images, basemaps, shapefiles, map data, etc.] in Figures 1, 2, 6, 7, 8, 9, 12, 13, 14 and 15?**

Thank you for your question.

The data named City boundary, District and Road network in Figures 1, 2, 6, 7, 8, 9, 12, 13, 14 were downloaded from OpenStreetMap (https://www.openstreetmap.org/), which is an open-source platform. We checked the journal's requirements for publication of figures, the data downloaded from the OpenStreetMap conforms to the Creative Commons Attribution (CC BY) license as long as the statement "Contains information from OpenStreetMap and OpenStreetMap Foundation, which is made available under the Open Database License.” (<https://journals.plos.org/plosone/s/figures>) We have added this statement to the title of the corresponding figures, as shown in the document named “Figure.docx”

The data named “Food outlets” in Figure 1 was obtained via the Baidu API (<http://lbsyun.baidu.com/index.php?title=webapi/guide/webservice-placeapi>), which is an open-source platform.

The estimated data in Figures 2, 6, 7, 8, 9, 12, 13, 14 and 15 were calculated by the methods of our study.

All the data we used to create maps was is uploaded to figshare at a private link (<https://figshare.com/s/267dbe734ba492ac41d6>), which has been explained in ***Data availability statement***. When we uploaded data on this website, the licence of data is selected as CC BY 4.0. therefore, our data conforms to CC BY 4.0, as shown in following figures. When the article is accepted, we will make this link public for everyone to download our data.

**b) Please state whether the [map/satellite images] have been previously copyrighted to your knowledge.**

Thank you for this question.

As we have explained in the question 4, all the figures are created by Lu Wang who is the first author of this paper. Therefore, we have sought permission to use the figures 1, 2, 6, 7, 8, 9, 12, 13, 14 and 15 from her. And we uploaded the document named “Permission form.pdf” as a submission file labeled with the file type “Other” in new submission.

**c) If any of the [map/satellite] images have been previously copyrighted, we require specific consent from the copyright holder to publish these images in PLOS ONE, under the CC BY 4.0 license. To seek permission from the copyright owner to publish your map figures under the specific Creative Commons Attribution License (CCAL), CC BY 4.0, please contact them with the following text and PLOS ONE Request for Permission form (**[**https://journals.plos.org/plosone/s/file?id=7c09/content-permission-form.pdf**](https://journals.plos.org/plosone/s/file?id=7c09/content-permission-form.pdf)**):**

Thank you for your reminder.

All the figures were created by Lu Wang who is the first author of this paper. Therefore, we do not have the [map/satellite] images which have been previously copyrighted.

**“I request permission for the open-access journal PLOS ONE to publish the maps in Figures 1, 2, 6, 7, 8, 9, 12, 13, 14 and 15 under the Creative Commons Attribution License (CCAL) CC BY 4.0 (http://creativecommons.org/licenses/by/4.0/). Please be aware that this license allows unrestricted use and distribution, even commercially, by third parties. Please reply and provide explicit written permission to publish these maps under a CC BY license.” Please upload the granted permission to the manuscript as an other file. In the figure caption of the copyrighted figure, please include the following text: “Republished from [ref] under a CC BY license, with permission from [name of publisher], original copyright [original copyright year].”**

Thank you for this reminder.

We have uploaded the document named “Permission form.pdf” as a submission file labeled with the file type “Other” in new submission.

Reviewer 1:

Comments to the Author:

This study evaluates the "food desert" phenomenon in Shanghai using a linear tessellation and network constrained kernel density methods. It is an interesting topic and also an important issue in China that has rarely been concerned. This work presents a certain level of novelty and significance. However, some contents are not very clear and the details should be clarified. Here are my comments, and I hope they would be helpful for improvement.

The responses to all comments are as follows:

Comments:

**1. The English is fluent but some expressions are weird to me. For example, in section 2.1, "numbering 12419 in total" could be presented in a simpler way like "We obtained 12,419 records of the food retail outlets via ....". In section 3.3, "average house price square meter" could be "average house price per square meter". Please ask a native speaker to polish this article and make a throughout check on such issues.**

Thank you for your corrections.

We have revised the two sentences you mentioned in this comment. The corresponding changes in the manuscripts are as follows:

We obtained 12,419 records of the food retail outlets via the Baidu API (http://lbsyun.baidu.com/index.php?title=webapi/guide/webservice-placeapi), including full-service supermarket chains, fresh markets, sea food stores, fruit and vegetable grocery stores (Su et al., 2017); (Lines 194-198)

*P_h_* is the average house price per square meter; (Lines 526-527)

In addition, our original manuscripts have been edited for proper English language, grammar, punctuation, spelling, and overall style by one or more of the highly qualified native English-speaking editors at AJE which is a professional polish website for papers.

We hope you are satisfied with all the modifications.

**2. Equation 1 seems to be unclear. Firstly,** **some varibles are repeatly defined in equation 1 and 2. Secondly, what is f_j means in this study? Is this variable equivalent to f^i (****speed-based weight). Why this weight is calculated as the ratio between the speed and l_e? In that sense** **L_i should equal to vi if there is only one f?** **In the experiment, L_i seems to represent the movement that an object could move in 1s, which is confused to me. Please clarify this.**

Thank you for these commons.

For the problem about “some varibles are repeatly defined in equation 1 and 2”, we have rewritten the equations 1 and 2 to help the readers understand them more clearly and deleted the repeated defined varible *l_e_*.

The corresponding changes in the manuscripts are as follows:

The form of partition length is:

$L_{i}=l_{e}\cdot f_{i}$ （1）

where $L_{i}$ is the length of basic unit for $i^{th}$ road; $l_{e}$ is the defined length; $f_{i}$ is the weight function. (Lines 249-252)

Therefore, the weight function $f_{i}$ can be represented as:

$f_{i}=\left\{\begin{matrix} \frac{v_{i}}{l_{e}}\begin{matrix} & v_{i}>0 \end{matrix} \\ \frac{v_{\min}}{l_{e}}\begin{matrix} & v_{i}=0 \end{matrix} \end{matrix}\right.$ （2）

where $v_{i}$ is the average speed (m/s) of the $i^{th}$road; and $v_{\min}$ is the minimum speed (m/s) in human travel. (Lines 255-258)

For the second problem, we are sorry that the equation 1 we expressed in the original manuscripts was not clear. We have rewritten the equations 1 and 2 to help the readers understand them more clearly, as shown above.

For the last problem about the definition of the speed-based weight function, the weight calculated by the ratio between the speed and *le* in order to ensure that the road network is divided into a series of isochronous basic units, where each basic unit represents 1 second. Therefore, *L_i_* is equal to *v_i_* with this definition of weight function *f_i_*, as you mentioned in common. This definition would simply the identification process of “food desert” with the network Kernel Density Estimation method. Because in the process of network kernel density calculation, it is not necessary to calculate the length of the road segments cumulatively, only the number of road segments need to be counted.

The corresponding explanation about the definition of weight function *f_i_* had been mentioned in the original manuscripts, as follows:

Based on this speed-based weight, the road network is divided into a series of isochronous basic units with varying lengths, where each basic unit represents 1 second. (Lines 263-264)

We hope you are satisfied with all the modifications and explanation.

**3. I dont think((s-c)/tau) is a good expression in equation 4, whilst k(x), x= (s-c)/tau might be more clear.**

Thank you for your suggestion.

We have modified the expression of equations 3 and 4. The corresponding changes in the revised manuscripts are as follows:

Applying all the advantages of the KDE method to calculate event accessibility, the general formula is expressed as:

 (3)

(Lines 278-280)

Therefore, a quartic function is adopted as the weight function in our experiments of the planar method and the network method, which is often utilized for density calculation. The formula is as follows:

$\begin{matrix} k(x)=\frac{3}{4}\text{（}1-x^{2}\text{）} \\ x=\frac{s-c_{i}}{\tau} \end{matrix}$ (4)

(Lines 337-341)

We hope you are satisfied with these modifications about equations 3 and 4.

**4. What do the street speed means in metro mode and how does it measures? Because the metro network should be another network rather that the road, it is hard to interpret the results in street-level on metro mode.**

Thank you for this common.

As shown in Fig 2(d), the speed of metro network with 14 lines were calculated by the average speed of the corresponding line, the speed of road network utilized the calculated speed of the bicycle mode. Because the metro lines are connected with the road lines. The behavior of residents going to the metro station to take the metro usually occurs in the road network. Therefore, we utilized the speed of bicycle mode to simulate the behavior of residents going to the metro station.

We hope you are satisfied with this explanation.

**5. In section 3.3, the conclusion "****the results from the network method show that two-thirds of the low-income residents are more likely to suffer from the “food desert”, whereas two-thirds of the high-income residents have a better food-access environment" could be problematic because i don' think the actual population distribution has been considered in this study. The statistic here is just the proportion of area rather than the real population number.**

Thank you for this correction.

We are sorry that the description of this conclusion is not accurate. We have modified it.

The corresponding changes in the manuscripts are as follows:

Comparing the proportion between patterns 1 and pattern 3 and the proportion between patterns 0 and pattern 2 in all modes, the results from the network method show that residents in two-thirds of low-income areas are more likely to suffer from the “food desert”, whereas residents in two-thirds of the high-income areas have a better food-access environment. (Lines 656-660)

We hope you are satisfied with this modification.

Minor comments:

**1. Section 2.2.1, please add some** **reference for Kalman filtering and state the benefit for using it here.**

Thank you for your suggestion.

We have added the references for Kalman filtering and stated its benefit in the revised manuscripts. The corresponding changes in the manuscripts are as follows:

The average speed of each road segment is calculated by trajectory data with Kalman filtering method (Kobayashi et al., 1995). Because Kalman filter can estimate the position and speed of a vehicle from a series of incomplete and noisy data, which makes it an optimal estimation method and is widely used in the field of automatic driving. (Lines 258-262)

The added reference is as follows:

Kobayashi, K., Cheok, KC., & Watanabe, K., 1995. Estimation of absolute vehicle speed using fuzzy logic rule-based Kalman filter. In: Proceedings of 1995 American Control Conference-ACC'95. IEEE, Vol. 5, pp. 3086-3090. (Lines 803-805)

We hope you are satisfied with the description of the Kalman filtering method and the corresponding modifications.

**2. The authors may want to add the name of travel mode as the title of every subplot in like figure 10, 11.**

Thank you for your suggestion.

We have added the title of every subplot in all revised figures. The original Figures 10 and 11 you mentioned in this common have been changed to Figures 13 and 14 in the revised manuscripts, respectively.

We hope you are satisfied with these modifications about figures.

Reviewer 2:

Comments to the Author:

The paper presents an in-depth analysis of food accessibility in urban and suburban areas, focusing on the phenomenon of "food deserts". It uses a combination of Kernel Density Estimation (KDE) and spatial overlay analysis to identify areas at risk of becoming a food desert. The paper also provides a comparative analysis of the planar and network methods in estimating the patterns of food accessibility. Further, the paper provides a nuanced understanding of the food desert phenomenon by defining four patterns of risk, which can be useful for policymakers and urban planners.

Strong points:

1. An innovative framework for identifying "food desert" areas, which is highly meaningful for urban planning and public health.

2. Real-world Application with different modes of transport and their impact on food accessibility.

3. A detailed classification of the "food desert" phenomenon with four patterns of risk, ranging from risk-free areas to top risk level areas, which are the actual "food deserts".

4. A comparative analysis of the planar and network methods in estimating the patterns of food accessibility.

Opportunities for improvement:

**1.** **The results in this paper are mostly statistical and rather abstract. It would improve the paper if the authors add some concrete case studies to demonstrate the effectiveness of the proposed method. In particular, it would be nice if the authors can show examples of different stages of food deserts.**

Thank you for your meaningful suggestion.

Traditional methods of assessing “food desert” phenomena are generally based on the planer space, which ignore the fact that human activities of acquiring food are restricted to street network space. To improve the above problems, this study proposes a linear tessellation model and designs the network kernel density estimation method and the network K-function analysis method based on this model to assess the food accessibility. The experiments using the planar methods with equivalent parameters are conducted to evaluate the validity of the network methods proposed in our study. We utilized a lot of statistical data in Section 3 to analyze the results of the experiments for three reasons:

(1) The differences of KDE value between the planar methods and the network methods are quantitatively evaluated to demonstrate that the traditional planar method overestimates food accessibility in terms of both range and value. This conclusion is described in paragraphs 1 and 2 of Section 3.1.

(2) The differences of KDE value among four transport modes are quantitatively evaluated to demonstrate that the proposed KDE method based on the linear tessellation model can not only reflect that food accessibility is affected by distance, which is similar with the traditional planer KDE method, but also reflect that food accessibility is susceptible to the real-time traffic condition and road planning, which cannot be evaluated by the traditional planer method. Therefore, we concluded that the proposed network KDE method has the ability to simulate human behavior of acquiring food along the road network in comparison with the planar method, as discussed in paragraphs 3 and 6 of Section 3.1.

(3) The differences of K-function result among four areas are quantitatively evaluated to demonstrate that food POIs are distributed evenly and reasonably in urban center areas, but clustered in suburb areas, which is the reason why food accessibility is high and balanced in the central urban areas but unbalanced in the suburb areas. This conclusion is described in Section 3.2.

In addition, the network KDE values and income values are utilized to comprehensively identify the risk degree of “food desert” phenomena in different regions. As shown in Section 3.3, firstly, we used statistical data to delineate four levels of risk pattern for food desert phenomena, with the risk increasing as the pattern index increases. Then, we analyzed the distribution of the four levels of risk pattern and draw conclusion that people in disadvantaged areas of Shanghai are more susceptible to negative food-access environments. To prove the validity of this conclusion, we have cited related literatures (Lines 579-582) and referred to the planning policy of Shanghai (Lines 588-590). However, there are few studies and related policies on the assessment of food desert phenomenon in Shanghai. Therefore, it is difficult for us to use many concrete case studies to prove the efficiency of our proposed methods. It is easier to compare the two KDE methods with statistical data, and it is more intuitive to show the difference between these two methods. In future research, we will continue to follow up on case studies of “food desert” phenomena in Shanghai.

The suggestion about “different stages of food deserts” is very meaningful. To analyze the food desert phenomena at different stages, it is necessary to obtain the GPS trajectory data, food POIs data, road network data, housing price data, etc., at each stage. It will take a certain amount of time to collect the data of different stages. Therefore, we considered this suggestion as our future work to refine our study. (Lines 701-703)

We hope you are satisfied with our explanation.

**2. The paper can benefit from analysis on the impact of different government policies or interventions aimed at improving food accessibility and reducing "food desert" phenomena.**

Thank you for your suggestion.

We totally agree with your idea. We have tried to find the policy proposed by government to address the "food deserts" phenomenon in the study region. Unfortunately, there is no special clause in Chinese urban planning that regulates the healthy food stores and targets at the food deserts, which results in no policy on this phenomenon.

As an alternative, we put forward some suggestions to improve the “food desert” phenomenon from the perspective of urban planning. In *Section 3.4*, based on the characteristics of the study region, from the two aspects of food retail POIs and transportation, we have summarized suggestions of planning that can alleviate the food deserts phenomenon to some extent. In terms of food retail POIs, the proposal is to develop the reasonable planning for the distribution and number of full-service healthy food outlets. There are three suggestions for transportation planning, including: (1) reasonable planning for the distribution and number of full-service healthy food outlets; (2) increasing the popularity of subway facilities; (3) increasing the number of shared bikes in city fringes; (4) “odd-and-even license plate rule” for the cars in urban areas.

We hope you are satisfied with the proposed suggestions of urban planning to improve the “food desert” phenomenon in *Section 3.4*.

**3. The authors apply specifically KDE and spatial overlay analysis. I'm curious if other data analysis methods can be used for the same purpose, such as clustering, graph analytics, and dimensionality reduction methods.**

Thank you for this comment.

We think that this suggestion makes a lot of sense. We have studied the data analysis methods mentioned in your comment. By analyzing, we draw the following conclusions:

The clustering is indeed commonly used to analyze geography data, and it conforms to the Tobler's first law of geography. However, it hardly reflects the trend of the “distance-decay effect” of the service capacity of food POIs. In addition, the speed at which humans move through the road network varies depending on the traffic conditions and the mode of transportation used for acquiring food, which also affects the ability to access food. The clustering method is difficult to take the above factors into account.

The purpose of our study was to delineate the scope of “food desert” problem. The graph analysis method focuses on the strength and direction of relationships between objects in a graph. Is the dimensionality reduction method a machine learning method you mentioned in this comment? If so, we do not understand how it applies to food accessibility analysis. Therefore, we concluded that these two methods are less applicable to analyze food accessibility in our study.

The network KDE method based on the linear tessellation model proposed in our study not only takes into account the “distance-decay effect” of the service capacity of food POIs, but also utilizes the speed calculated from the trajectory big data of different transportation modes to simulate the real state of human with different modes of transportation in the road network, thus simulating the human behaviors of acquiring food for a better assessment of food accessibility.

The advantages of the network kernel density method have been described in detail in the chapter *1.2 Literature review.* And the reasons why we proposed the Network Kernel Density Estimation method based on the linear tessellation and utilized it to analyze the “food desert” phenomenon have been introduced in the chapter *1.3 Current study*, aiming to combine the advantage of the scalability of the geographic grid with the advantage of human behavior simulation of the network KDE method.

We hope you are satisfied with our explanation of the choice of network kernel density analysis method.

**4. Finally, the study can be enriched by also analyzing the temporal aspects of the data, e.g., a longitudinal study showing** **how an area deteriorates into a food desert over time, and how different classes of food deserts can gradually transform into other classes.**

Thank you for this meaningful suggestion.

This is really a meaningful study to analyze the temporal aspects of the data, which provides a new perspective of analyzing the “food desert” problem and adds dimension to our study. We would like to try the study you mentioned in this comment in the future research. There are the following reasons.

First, the focus of this study is to explore the effectiveness of the proposed Network Kernel Density Estimation method based on the linear tessellation in analyzing food desert phenomena by comparing with the traditional planar Kernel Density Estimation method. Therefore, our research pays more attention to the innovation of spatial data model and algorithm.

Second, the temporal aspect of the data needs to be analyzed not only by obtaining trajectory data as well as food retail outlet data for the same time period, but also for multiple time periods. Therefore, it is difficult to obtain data that fully meets the above requirements. We need more time to collect data from websites or contact the appropriate agency to get the data.

For the above reasons, we took your suggestions as the direction of our future research. The corresponding texts were added in the chapter *4.3 Limitations*, as follows:

Thirdly, we analyzed the “food desert” phenomenon using data from only one time period (May 2017), lacking data from different time periods to explore that how an area deteriorates into a food desert over time. (Lines 701-703)

We will utilize data from more time periods and combine socioeconomic variables and data to analysis the “food desert” phenomenon in more depth. (Lines 708-710)

We hope you are satisfied with our explanation and the corresponding changes in the revised manuscript.

I suggest that the authors carefully revise the paper to address the above points.

Minor issues:

**1. The phrase "the quantity and distribution of food outlets satisfy the majority of residents’ demands for food access considering the aspect of distance but without considering the real behaviour of acquiring" could be rephrased for clarity. It's not entirely clear what "****the real behaviour of acquiring" refers to.**

Thank you for this correction.

We are sorry that the expression is not clear. The “the real behaviour of acquiring” refers to the process by which residents acquire food in the road networks. The planar KDE method is calculated by the Euclidean distance. However, the process of acquiring food occurs in the road networks. Therefore, the estimated results of the planar KDE method can not simulate the real behavior of acquiring food.

To make readers understand this statement more clearly, we have modified the expression of this sentence. The corresponding changes in the manuscripts are as follows:

This shows that the identified risk level is much lower when the planar KDE method is applied; that is to say, the quantity and distribution of food outlets satisfy the majority of residents’ demands for food access considering the aspect of distance but without taking into account the fact that the real process of acquiring food occurs in the road network. (Lines 568-571)

We hope you are satisfied with this modification.

**2. The sentence "Furthermore, the heavy traffic aggravates their inaccessibility" could be more specific. It's not clear whose inaccessibility is being referred to.**

Thank you for this correction.

We are sorry that this expression is not specific. The “their inaccessibility” refers to the inaccessibility of residents living in fringe areas. We have modified it to help the readers understand this sentence more clearly.

The corresponding changes in the manuscripts are as follows:

Furthermore, the heavy traffic aggravates the food inaccessibility of residents living in fringe areas. (Lines 578-579)

We hope you are satisfied with this modification.

**3. The phrase "the even distribution of food outlets along the roads determines the widespread accessibility in all modes except for the areas with heavy traffic jams" could be rephrased for clarity. It might be clearer to say something like "the even distribution of food outlets along the roads ensures widespread accessibility in all modes, except in areas with heavy traffic jams".**

Thank you for this suggestion.

We are sorry that this expression is not clear. We have modified it according to your suggestion.

The corresponding changes in the manuscripts are as follows:

In the results of planar method, the clustered but adequate food retails ensure that the residents demand for food will be met. In addition, in the results of the network method, the even distribution of food outlets along the roads ensures widespread accessibility in all modes, except in areas with heavy traffic jams. (Lines 583-586)

We hope you are satisfied with this modification.
