# Supplementary material for: A linear tessellation model for the identification of “food desert”: A case study of Shanghai, China
Source: PLoS One. 2025 Jan 22;20(1):e0317003. doi: 10.1371/journal.pone.0317003 (PMC11753656; doi:10.1371/journal.pone.0317003)
Supplement: Attachment — Submitted filename: Response to Reviewers.docx [file pone.0317003.s002.docx]

Responses

We thank the reviewers for their valuable comments, which have enabled us to significantly improve this paper. We carefully revised the manuscripts and corresponding figures. We chose the track changes mode in Word to highlight our changes in the manuscripts, where the texts in blue and strikethrough refers to the deleted contents, and the texts in red and underline refers to the added contents. The line number mentioned is the revised manuscript without changes marked.

Please find our responses to each individual comment below.

Journal Requirements:

Please review your reference list to ensure that it is complete and correct. If you have cited papers that have been retracted, please include the rationale for doing so in the manuscript text, or remove these references and replace them with relevant current references. Any changes to the reference list should be mentioned in the rebuttal letter that accompanies your revised manuscript. If you need to cite a retracted article, indicate the article’s retracted status in the References list and also include a citation and full reference for the retraction notice.

Thank you for your requirement.

According to a comment from reviewer 1, he/she thinks there are too many references in our paper, and some of them are ever redundant. Therefore, we remove some redundant references, and the number of references is reduced from 90 to 72 in the revised manuscripts.

The removed 18 references are as follows:

[1] Apparicio, P., Cloutier, M. & Shearmur, R., 2007. The case of Montréal's missing food deserts: Evaluation of accessibility to food supermarkets. Int. J. Health Geogr. 6(4), 1-13.
[2] Bailey, T. C., Gatrell, A.C., 1995. Interactive spatial data analysis. New York, NY: John Wiley and Sons.
[3] Bailey, T.C., Gatrell, A.C., 1995. Interactive Spatial Data Analysis. Longman Scientific, Harlow, UK.
[4] Basu, S., & Vasudevan, V., 2013. Effect of bicycle friendly roadway infrastructure on bicycling activities in urban India. Part of Special Issue: 2nd Conference of Transportation Research Group of India. P. Chakroborty, H.K. Reddy and A. Amekudzi (eds.). Procedia – Social and Behavioral Sciences. 104, 1139-1148.
[5] Bell, J., Mora, G., Hagan, E., et al., 2013. Access to healthy food and why it matters: A review of the research. Philadelphia, PA: The Food Trust.
[6] Bureau, S. S., 2018. Shanghai statistical bulletin on national economic and social development. Statistical science and practice. 03, 11-21.
[7] Cummins, S., & Macintyre, S., 2002. “Food deserts”—evidence and assumption in health policy making. BMJ. 325(7361), 436-438.
[8] Cummins, S., 2014. Food deserts. The Wiley Blackwell Encyclopedia of Health, Illness, Behavior, and Society.
[9] Epanechnikov, V. A., 1969. Non-parametric estimation of a multivariate probability density. Theory Probab. Appl. 14(1), 153-158.
[10] Kobayashi, K., Cheok, KC., & Watanabe, K., 1995. Estimation of absolute vehicle speed using fuzzy logic rule-based Kalman filter. In: Proceedings of 1995 American Control Conference-ACC'95. IEEE, Vol. 5, pp. 3086-3090.
[11] Nie, K., Wang, Z., Du, Q., et al., 2015. A network-constrained integrated method for detecting spatial cluster and risk location of traffic crash: A case study from Wuhan, China. Sustainability. 7(3), 2662-2677.
[12] Rhone, A., Ver Ploeg, M., Dicken, C., et al., 2017. Low-income and Low-supermarket-access Census Tracts, 2010-2015. Washington, DC: US Department of Agriculture, Economic Research Service.
[13] Silverman, B.W., 1986. Density Estimation for Statistics and Data Analysis. Chapman Hall, London, UK.
[14] Wang, M., 2018. Exploring the direction of “city-oriented policy” in key cities from the perspective of “price-to-income ratio deviation”. Shanghai Real Estate. 376(06):14-17.
[15] Wang, S., Zhang, J., Liu, L., et al., 2010. Bike-Sharing-A new public transportation mode: State of the practice & prospects. In: Emergency Management and Management Sciences (ICEMMS), 2010 IEEE International Conference on, pp. 222e225
[16] Wang, S., Zhang, J., Liu, L., et al., 2010. Bike-Sharing-A new public transportation mode: State of the practice & prospects. In: Proceedings of 2010 IEEE International Conference on Emergency Management and Management Sciences, ICEMMS 2010, Beijing, China, August 8, 2010 - August 10, 2010, pp. 222-225.
[17] Wrigley, N., Warm, D., & Margetts, B., 2003. Deprivation, diet, and food-retail access: Findings from the Leeds ‘food deserts' study. Environ. Plan. A. 35(1), 151-188.
[18] Xie, Z., Yan, J., 2013. Detecting traffic accident clusters with network kernel density estimation and local spatial statistics: an integrated approach. J. Transp. Geogr. 31, 64-71.
[19] Zhang, T. W., 2002. Urban development and a socialist pro-growth coalition in shanghai. Urban Aff. Rev. 37(4), 475-499.

In addition, we have completed and corrected all the references. And we numbered the references in the order in which they were cited and inserted the numbers in the corresponding places.

We hope you are satisfied with all the modifications of references.

Comments from Reviewers:

Review Comments to the Author

Please use the space provided to explain your answers to the questions above. You may also include additional comments for the author, including concerns about dual publication, research ethics, or publication ethics. (Please upload your review as an attachment if it exceeds 20,000 characters)

The responses to all comments are as follows:

Reviewer 1:

Comments to the Author:

The author has essentially addressed the issues I raised last time. I believe that the overall technical approach and conclusions of this article are not problematic, but there are still some areas that need improvement in terms of writing.

The responses to all comments are as follows:

Comments:

**1. In the abstract and on line 65, the author specifically mentions the US. Since the subsequent text does not focus on the issue of food deserts in the US, it would be more appropriate to use “in many countries worldwide” here.**

Thank you for your corrections.

We have changed “in the United States and other countries” to “in many countries worldwide” in the revised manuscripts. (Lines 63-64)

**2. On line 80, the term “Density” requires clarification. What density is being referred to? The author needs to be more specific.**

Thank you for this suggestion.

We have a more specific explanation of density-based methods in the revised manuscripts. The corresponding changes are as follows:

The density-based methods quantify the accessibility of food outlets with total count, count per population or count per square area using the buffer method, kernel density estimation (KDE) or spatial clustering. (Lines 75-77)

We hope you are satisfied with all the modifications and explanation.

**3.** **On line 93, there is no need to quote Professor Tobler's original statement.**

Thank you for your suggestion.

We have deleted the Professor Tobler's original statement of Tobler's First Law in the revised manuscripts. (Line 86)

**4. On line 96, the k-function is introduced. This can be presented as a separate paragraph since it is quite different from the previous contents.**

Thank you for this common.

According to your suggestion, the introduction of the k-function has been as a separate paragraph in the revised manuscripts. (Line 90)

**5. On line 148, what does it mean by “current study”? Is there a significant difference between this and a literature review?**

Thank you for this question.

The section 1.3 Current study refer to our study objective of this paper. To make this section clearer for readers, we changed the title to “Study objective” in the revised manuscripts. (Line 136)

**6. On line 186, abbreviations like POI should be accompanied by their full name the first time they appear.**

Thank you for this correction.

We have added the full name of POIs in the revised manuscripts. (Line 171)

**7. On line 193, the full name of SODA might be “Shanghai Open Data Apps”, and at least should not include “competition.”**

Thank you for this correction.

We have corrected the full name of SODA as “Shanghai Open Data Apps” in the revised manuscripts. (Line 178)

**8. From line 192 to 199, the information about these websites can be inserted as footnotes in the document.**

Thank you for your suggestion.

The corresponding the URL has been inserted as footnotes in the revised manuscripts. (Lines 167-182)

**9. For Figures 14 and 15, I do not think using “pattern 0-4” is a good idea. Firstly, there is no “pattern 2” here, which seems odd. Moreover, using numbers does not convey the actual meaning of the patterns. You can refer to the commonly used expressions in GIS, such as LL (low-low), to express the patterns you are interested in.**

Thank you for this comment.

According to your suggestion, we added the pattern name to express four patterns. Pattern name HH expresses pattern of the higher accessibility and higher income. Pattern name HL expresses pattern of the higher accessibility and lower income. Pattern name LH expresses pattern of the low accessibility and higher income. Pattern name LL expresses pattern of the low accessibility and low income. The original pattern index is changed to risk level in the revised manuscripts and Table 3. We have added explanation of the corresponding modification. The changes are as follows:

Finally, we can obtain four patterns with cross combination, as shown in Table 4. The four patterns are named as HH, HL, LH and LL, corresponding to the risk level 0, 1, 2 and 3, respectively, where the higher risk level represents the higher risk of “food desert”. (Lines 494-496)

In addition, we have changed patterns 0, 1, 2 and 3 to patterns HH, HL, LH and LL, respectively, in the revised manuscripts and Figures 14 and 15.

We hope you are satisfied with all the modifications.

**10. There are too many references in this article, and some of them are ever redundant. For example, it might not be necessary to cite ten references to demonstrate the importance of food accessibilty to low income population without reviewing in their details.**

Thank you for this suggestion.

We have removed the redundant references. The number of references has been reduced from 90 to 72 in the revised manuscripts.

The removed 18 references are as follows:

[1] Apparicio, P., Cloutier, M. & Shearmur, R., 2007. The case of Montréal's missing food deserts: Evaluation of accessibility to food supermarkets. Int. J. Health Geogr. 6(4), 1-13.
[2] Bailey, T. C., Gatrell, A.C., 1995. Interactive spatial data analysis. New York, NY: John Wiley and Sons.
[3] Bailey, T.C., Gatrell, A.C., 1995. Interactive Spatial Data Analysis. Longman Scientific, Harlow, UK.
[4] Basu, S., & Vasudevan, V., 2013. Effect of bicycle friendly roadway infrastructure on bicycling activities in urban India. Part of Special Issue: 2nd Conference of Transportation Research Group of India. P. Chakroborty, H.K. Reddy and A. Amekudzi (eds.). Procedia – Social and Behavioral Sciences. 104, 1139-1148.
[5] Bell, J., Mora, G., Hagan, E., et al., 2013. Access to healthy food and why it matters: A review of the research. Philadelphia, PA: The Food Trust.
[6] Cummins, S., & Macintyre, S., 2002. “Food deserts”—evidence and assumption in health policy making. BMJ. 325(7361), 436-438.
[7] Cummins, S., 2014. Food deserts. The Wiley Blackwell Encyclopedia of Health, Illness, Behavior, and Society.
[8] Epanechnikov, V. A., 1969. Non-parametric estimation of a multivariate probability density. Theory Probab. Appl. 14(1), 153-158.
[9] Kobayashi, K., Cheok, KC., & Watanabe, K., 1995. Estimation of absolute vehicle speed using fuzzy logic rule-based Kalman filter. In: Proceedings of 1995 American Control Conference-ACC'95. IEEE, Vol. 5, pp. 3086-3090.
[10] Nie, K., Wang, Z., Du, Q., et al., 2015. A network-constrained integrated method for detecting spatial cluster and risk location of traffic crash: A case study from Wuhan, China. Sustainability. 7(3), 2662-2677.
[11] Rhone, A., Ver Ploeg, M., Dicken, C., et al., 2017. Low-income and Low-supermarket-access Census Tracts, 2010-2015. Washington, DC: US Department of Agriculture, Economic Research Service.
[12] Silverman, B.W., 1986. Density Estimation for Statistics and Data Analysis. Chapman Hall, London, UK.
[13] Wang, M., 2018. Exploring the direction of “city-oriented policy” in key cities from the perspective of “price-to-income ratio deviation”. Shanghai Real Estate. 376(06):14-17.
[14] Wang, S., Zhang, J., Liu, L., et al., 2010. Bike-Sharing-A new public transportation mode: State of the practice & prospects. In: Emergency Management and Management Sciences (ICEMMS), 2010 IEEE International Conference on, pp. 222e225
[15] Wang, S., Zhang, J., Liu, L., et al., 2010. Bike-Sharing-A new public transportation mode: State of the practice & prospects. In: Proceedings of 2010 IEEE International Conference on Emergency Management and Management Sciences, ICEMMS 2010, Beijing, China, August 8, 2010 - August 10, 2010, pp. 222-225.
[16] Wrigley, N., Warm, D., & Margetts, B., 2003. Deprivation, diet, and food-retail access: Findings from the Leeds ‘food deserts' study. Environ. Plan. A. 35(1), 151-188.
[17] Xie, Z., Yan, J., 2013. Detecting traffic accident clusters with network kernel density estimation and local spatial statistics: an integrated approach. J. Transp. Geogr. 31, 64-71.
[18] Zhang, T. W., 2002. Urban development and a socialist pro-growth coalition in shanghai. Urban Aff. Rev. 37(4), 475-499.
[19] Zhang, T. W., 2002. Urban development and a socialist pro-growth coalition in shanghai. Urban Aff. Rev. 37(4), 475-499.

We hope you are satisfied with our modifications.

**11. Figure 3, please keep the font and style consistent in figures. For example, some words are relatively too small in figure 3 and the first-letter vary between uppercase and** **lowercase. Similar issues also exist in Figure 4. it will be better if "the result of..." could be "The result of ...", "projection point" could be "Projection point"....**

Thank you for this correction.

To keep the font and style consistent in figures, the first letter of words in Figures 3 and 4 has been changed to uppercase. The subtitles of each figure are lowercase. In addition, the words in Figure 3 have been enlarged. All the changes are as seen in the document named “Figure.doc” and the submitted figures in the document named “Figures.rar”

We hope you are satisfied with these modifications.

**12. The unit for distance measurement should be consistent throughout the manuscipt. In Figure 11, the distance unit is meter, while in figure 13, for instance, the unit is mile.**

Thank you for this comment.

We have standardized the distance measurement to kilometer throughout all the figures, as seen in the document named “Figure.doc” and the submitted figures in the document named “Figures.rar”.

We hope you are satisfied with all modifications of figures.

Reviewer 3:

Comments to the Author:

**1. Conclusions Should be supported by data, kindly add data to support the conclusions with KDE values and K Function Curves.**

Thank you for your meaningful suggestion.

We have added the data to describe the differences of KDE values and K-function results between planer and network space in section Conclusion to support findings of our study. The corresponding changes are as follows:

The KDE values of the planar method are greater than those of the network method, especially in walk and metro modes, where the values of the planar results are nearly **three times higher** than those of the network results. The planar KDE results have larger scope than the network KDE results, which is obvious in bicycle and taxi modes, as evidenced by the fact that the scopes of the planar results are **almost twice as large as** those of the network results. These differences between two KDE methods under four transport modes indicate that food accessibility is not only related to the distance to food outlets, but also related to the actual travel constraints encountered in the process of acquiring food, such as real-time traffic conditions, road planning and the accessibility of transport facilities. The cumulative number of points in the results of the K-function method within planar space is **more than ten times higher** than that within network space at the same distance. According to these differences of results of the K-function method between two spaces within urban centers, urban fringes, suburbs and whole city areas, we analysis the reasons of unbalanced food accessibility from the perspective of food outlets’ distribution pattern. (Lines 604-617)

We hope you are satisfied with our modifications.

**2. No References should be added in conclusion section so remove all in text references from conclusion section.**

Thank you for your correction.

We have removed all citations from Conclusion section. And the corresponding references are removed from Reference section.

We hope you are satisfied with this revision.
