# Supplementary material for: A linear tessellation model for the identification of “food desert”: A case study of Shanghai, China
Source: PLoS One. 2025 Jan 22;20(1):e0317003. doi: 10.1371/journal.pone.0317003 (PMC11753656; doi:10.1371/journal.pone.0317003)
Supplement: Attachment — Submitted filename: Response to Reviewers.docx [file pone.0317003.s003.docx]

Responses

We thank the reviewers for their valuable comments, which have enabled us to significantly improve this paper. We carefully revised the manuscripts. We chose the track changes mode in Word to highlight our changes in the manuscripts, where the texts in blue and strikethrough refers to the deleted contents, and the texts in red and underline refers to the added contents. The line number mentioned is the revised manuscript without changes marked.

Please find our responses to each individual comment below.

Journal Requirements:

**1.Please review your reference list to ensure that it is complete and correct. If you have cited papers that have been retracted, please include the rationale for doing so in the manuscript text, or remove these references and replace them with relevant current references. Any changes to the reference list should be mentioned in the rebuttal letter that accompanies your revised manuscript. If you need to cite a retracted article, indicate the article’s retracted status in the References list and also include a citation and full reference for the retraction notice.**

Thank you for your notice.

According to comments from reviewer in the last revision, we have removed some redundant references and checked all the remained references to ensure the completeness and accuracy of references.

**2. When completing the data availability statement of the submission form, you indicated that you will make your data available on acceptance. We strongly recommend all authors decide on a data sharing plan before acceptance, as the process can be lengthy and hold up publication timelines. Please note that, though access restrictions are acceptable now, your entire data will need to be made freely accessible if your manuscript is accepted for publication. This policy applies to all data except where public deposition would breach compliance with the protocol approved by your research ethics board. If you are unable to adhere to our open data policy, please kindly revise your statement to explain your reasoning and we will seek the editor's input on an exemption. Please be assured that, once you have provided your new statement, the assessment of your exemption will not hold up the peer review process.**

Thank you for your requirement.

The link showed in Data availability statement was dead. We have shared our data via Github. The new link of our data is public for everyone, which is <https://github.com/wl7/A-linear-tessellation-model-for-the-identification-of-food-desert-A-case-study-of-Shanghai-China>.

Comments from Reviewer 3:

The responses to all comments are as follows:

Comments:

**1. Title of Section 1.3 Should be Objectives of the Study and it should be followed by section on Limitation of the study which now is part of conclusions section**

Thank you for your corrections.

Section 1.3 was intended to introduce the purpose of this study to the readers. We feel sorry about the inappropriate descriptions of this section. To make it clearer for readers to understand our study purpose and contents, we revised the texts in the manuscripts. The corresponding changes are as follows:

Based on the proposed linear tessellation model, this study attempts to: (1) introducing the network Kernel Density Estimation analysis method into the linear tessellation model to measure transit-varying food accessibility in Shanghai with trajectory data; (2) applying the network K-function method to analyze the estimated food accessibility from the perspective of food outlets’ distribution pattern; (3) identifying the "food desert" areas by the food accessibility and income and discussing the implications for urban planning The proposed model aims to provide an innovative thought with a rational analysis space for food acquirement issue, and to provide reasonable implications for public policymakers by exploring the state of “food desert” at a finer size. (Lines 147-154)

We hope you are satisfied with these modifications.

**2. Line 635-642 in Conclusion is part of introduction and is repetition in summary**

Thank you for your comment.

We have deleted the part of the conclusion that duplicates the text in summary. And the corresponding changes in the revised manuscripts are as follows:

This paper proposed a linear tessellation model to simulate activities of food acquisition within road networks and applies it to identify the “food desert” phenomenon of walk, bicycle, taxi and metro mode within **a city of developing country, namely Shanghai, China.** (Lines 596-598)

We hope you are satisfied with these modifications.

**3. Write conclusion in present perfect tense (presently written in present tense).**

Thank you for your correction.

We have revised the tense of section Conclusion according to the description of each sentence. For example, the sentences describing research results are in past tense or in present perfect tense, the sentences describing research significance are in present tense, the sentences describing research prospect are in future tense.

We hope you are satisfied with these modifications.

**4. Conclusions should be supported by data from analysis, write values to support three times in line 645, and ten times line 653**

Thank you for your suggestion.

We have added the experimental data to support three times and ten times in the conclusion. The corresponding are as follows:

The KDE values of the planar method were greater than those of the network method, especially in walk and metro modes, where the KDE value ranges were 7.7-1973.0 and 8.0-2049.2 in the planar results and they were 0.6-567.5 and 2.3-667.5 in the network results. The greatest KDE values of the planar results were nearly three times higher than those of the network results in walk and metro modes. (Lines 600-604)

The planar KDE results had larger scope than the network KDE results, which was obvious in bicycle and taxi modes, as evidenced by the fact that the scopes of the planar results with 90.6% and 84.0% were almost twice as large as those of the network results with 50.5% and 47.3%. (Lines 604-607)

The cumulative number of points in the results of the K-function method within planar space was more than ten times higher than that within network space at the same distance. Taking the K-function results of urban center region for instance, the cumulative number of points at 4.5km was around 250 in the planar method but it was 13 in the network method. (Lines 610-614)

We hope you are satisfied with these modifications.

**5. Innovation points Section Should also be written in Present perfect tense.**

The responses are same as question 3.
