# Supplementary material for: A linear tessellation model for the identification of “food desert”: A case study of Shanghai, China
Source: PLoS One. 2025 Jan 22;20(1):e0317003. doi: 10.1371/journal.pone.0317003 (PMC11753656; doi:10.1371/journal.pone.0317003)
Supplement: Attachment — Submitted filename: Response to Reviewers.docx [file pone.0317003.s004.docx]

Responses

We thank the reviewers for their valuable comments, which have enabled us to significantly improve this paper. We carefully revised the manuscripts. We chose the track changes mode in Word to highlight our changes in the manuscripts, where the texts in blue and strikethrough refers to the deleted contents, and the texts in red and underline refers to the added contents. The line number mentioned is the revised manuscript without changes marked.

Please find our responses to each individual comment below.

Journal Requirements:

**1.** **Please review your reference list to ensure that it is complete and correct. If you have cited papers that have been retracted, please include the rationale for doing so in the manuscript text, or remove these references and replace them with relevant current references. Any changes to the reference list should be mentioned in the rebuttal letter that accompanies your revised manuscript. If you need to cite a retracted article, indicate the article’s retracted status in the References list and also include a citation and full reference for the retraction notice.**

Thank you for your notice.

According to comments from reviewer in the last revision, we have removed some redundant references. This time, we checked all the remained references and revised references 1, 2, 8, 42, 54, 58, 59, 62, 67 to make them complete and correct.

We hope you are satisfied with these modifications.

Comments from Reviewer 3:

The responses to all comments are as follows:

Comments:

**1. Add Limitations Section after objectives as communicated earlier.**

Thank you for this comment.

The comment you made in the last revision is “Title of Section 1.3 Should be Objectives of the Study and it should be followed by section on Limitation of the study which now is part of conclusions section”.

In our last response, we have revised the context of Section 1.3 as follows:

Based on the proposed linear tessellation model, this study attempts to: (1) introducing the network Kernel Density Estimation analysis method into the linear tessellation model to measure transit-varying food accessibility in Shanghai with trajectory data; (2) applying the network K-function method to analyze the estimated food accessibility from the perspective of food outlets’ distribution pattern; (3) identifying the "food desert" areas by the food accessibility and income and discussing the implications for urban planning The proposed model aims to provide an innovative thought with a rational analysis space for food acquirement issue, and to provide reasonable implications for public policymakers by exploring the state of “food desert” at a finer size. (Lines 147-154)

We apologize for not explaining this revision well enough in our last response. In Section 1.2 Literature review, we summarized the problems that exist in the current researches on the analysis of food deserts phenomenon. Therefore, in Section 1.3, we present that our study intends to propose a linear tessellation model to address the problems summarized in Section 1.2, and we simply listed three study objectives to be achieved based on the proposed linear tessellation model. The context of subsequent Section 2 and 3 mainly focused on the linear model and three study objectives.

The Section 4.3 Limitations you mentioned in this comment is summarized based on the experimental results of this study and provides directions for future research. If Section 4.3 Limitations is placed after Section 1.3 Study objectives, readers may be confused about Section 4.3 because they have not yet understood the methods and experiments.

Therefore, in our last revision, we modified the contents of Section 1.3, and Limitations wad still placed in the Section 4 conclusions, which aims to make the article logic smoother. To more accurately express the content of section 4.3, the title of this section is changed as “Limitation and future researches”.

We hope you are satisfied with our explanation.

**2. Revise the section on Objectives as required.**

Thank you for this comment.

In our last response, we have revised Section 1.3 Study objective to make it clearer for readers to understand our study purpose and contents. The corresponding changes are reflected in the response to question 1.
