# Supplementary material for: A linear tessellation model for the identification of “food desert”: A case study of Shanghai, China
Source: PLoS One. 2025 Jan 22;20(1):e0317003. doi: 10.1371/journal.pone.0317003 (PMC11753656; doi:10.1371/journal.pone.0317003)
Supplement: Attachment — Submitted filename: Response to Reviewers.docx [file pone.0317003.s005.docx]

Responses

We thank the reviewers for their valuable comments, which have enabled us to significantly improve this paper. We carefully revised the manuscripts. We chose the track changes mode in Word to highlight our changes in the manuscripts, where the texts in blue and strikethrough refers to the deleted contents, and the texts in red and underline refers to the added contents. The line number mentioned is the revised manuscript without changes marked.

Please find our responses to each individual comment below.

Journal Requirements:

**1.** **Please review your reference list to ensure that it is complete and correct. If you have cited papers that have been retracted, please include the rationale for doing so in the manuscript text, or remove these references and replace them with relevant current references. Any changes to the reference list should be mentioned in the rebuttal letter that accompanies your revised manuscript. If you need to cite a retracted article, indicate the article’s retracted status in the References list and also include a citation and full reference for the retraction notice.**

Thank you for your notice.

We have corrected the references in the last revision.

**2. Please ensure that you include a legends for Figure 1 and 2 within your main document. We do appreciate that you have a figure legends document uploaded as a separate file, however, we do require this to be part of the manuscript file itself and not uploaded separately.**

Thank you for your suggestion.

We have added the legends of Figs 1 and 2 in our revised manuscripts.

The changes are as follows: (Lines 161-185)

Shanghai’s rapid urbanization and expansion have been affecting the communication, travel, work and lifestyles of millions of residents. It displays the positive and negative outcomes of urban growth, which makes it a typical place for diverse urban research [57].

To apply the proposed method to identify the food accessibility in the Shanghai region as shown in Fig 1(a), where the urban center and urban fringe area is within the red line and blue line, respectively, the remaining area is suburb.

The study data are as follows:

(1) Healthy food retail outlets. We obtained 12,419 records of the food retail outlets via the Baidu API, including full-service supermarket chains, fresh markets, sea food stores, fruit and vegetable grocery stores [13], as shown in the green points in Fig 1(b);

(2) Entire road networks. These data are downloaded from OpenStreetMap, as shown in the grey lines in Fig 1(b);

(3) House price Point of Interests (POIs). These data are obtained from the houses put up for sale in the Lianjia website in March, 2017. The total number of points is 91,619.

(4) Trajectory data. Allowing for the travel mode of residents and low ownership rate for private cars, four types of trajectory data, walk, bicycle, metro and taxi, are selected to calculate the average speed of each road segment for the corresponding travel mode. The estimated speed of the walk, bicycle, taxi and metro mode is shown in Fig 2(a), (b), (c) and (d), respectively.

GPS trajectory data of walk and bicycle are obtained from Yidong GPS APP. GPS trajectory data for taxi are obtained from SODA (Shanghai Open Data Apps). All the GPS trajectory data are in the same time period (March 1 to 31, 2017). The speed of metro mode is set based on the average speed of 14 metro lines in Shanghai, as shown in the illustration in the lower right corner of Fig 1(b). The average speed of 14 metro lines is calculated according to the total metro lines’ length provided by the Shanghai Survey and Mapping Institute^[1]^ and the total metro operation time provided by the Shanghai Metro official website.

**3. Can you please upload an additional copy of your revised manuscript that does not contain any tracked changes or highlighting as your main article file. This will be used in the production process if your manuscript is accepted. Please amend the file type for the file showing your changes to Revised Manuscript w/tracked changes. Please follow this link for more information:** [**http://blogs.PLOS.org/everyone/2011/05/10/how-to-submit-your-revised-manuscript/**](http://blogs.PLOS.org/everyone/2011/05/10/how-to-submit-your-revised-manuscript/)

Thank you for this notice.

We have uploaded the revised manuscript without tracked changes in the submission system.

**4. Please ensure that you include a legends/captions for Figure 1 and 15 within your main document.**

Thank you for this requirement.

We have added all the figures and their captions in the main document.

Comments from Reviewer 3:

The responses to all comments are as follows:

Comments:

**1. Check the grammar in the title of Section 4.3, Its title should be Limitations and Future Research.**

Thank you for your correction.

We have changed the title of Section 4.3 as “Limitations and Future Research” in the revised manuscripts. (Line 655)

We hope you are satisfied with our modification.

**2. Remove References from Objectives Section they should be part of literature review section and objectives should be framed by you based on the research gap identified after the literature review.**

Thank you for this comment.

We have moved the first paragraph of Section 1.3 to the end of Section 1.2. There are no references in the remained content of Section 1.3.

We hope you are satisfied with this modification.

**3. Paragraph one in Section 1.3 should be part of literature review and paragraph 2 of section should only be part of objectives section.**

Thank you for this comment.

We have moved the first paragraph of Section 1.3 to the end of Section 1.2 according to your suggestion.

We hope you are satisfied with this revision.
